# Anti-Inflammatory Potential and Synergic Activities of *Eclipta prostrata* (L.) L. Leaf-Derived Ointment Formulation in Combination with the Non-Steroidal Anti-Inflammatory Drug Diclofenac in Suppressing Atopic Dermatitis (AD)

**DOI:** 10.3390/life15010035

**Published:** 2024-12-30

**Authors:** Muhammad M. Poyil, Mohammed H. Karrar Alsharif, Mahmoud H. El-Bidawy, Salman Bin Dayel, Mohammed Sarosh Khan, Zainab Mohammed M. Omar, Alaaeldin Ahmed Mohamed, Reda M. Fayyad, Tarig Gasim Mohamed Alarabi, Hesham A. Khairy, Nasraddin Othman Bahakim, Mohamed A. Samhan, Abd El-Lateef Saeed Abd El-Lateef

**Affiliations:** 1 Department of Basic Medical Sciences, College of Medicine, Prince Sattam bin Abdulaziz University, Al-Kharj 11942, Saudi Arabia; m.elbidawy@psau.edu.sa (M.H.E.-B.); mo.khan@psau.edu.sa (M.S.K.); z.omar@psau.edu.sa (Z.M.M.O.); n.bahakim@psau.edu.sa (N.O.B.); as.ismail@psau.edu.sa (A.E.-L.S.A.E.-L.); 2 Department of Physiology, Faculty of Medicine, Cairo University, Kasr Al-Aini, Cairo 11956, Egypt; 3 Department of Internal Medicine, College of Medicine, Prince Sattam bin Abdulaziz University, Al-Kharj 11942, Saudi Arabia; s.bindayel@psau.edu.sa; 4 Department of Pharmacology, Faculty of Medicine, Al-Azhar University, Assiut 71524, Egypt; 5 Department of Pharmacology, Faculty of Medicine, Al-Azhar University, Damietta 34517, Egypt; 6 Department Pharmacology, General Medicine Practice Program, Batterjee Medical College, Asser 61961, Saudi Arabia; drredafayyadccmorc@gmail.com; 7 Department of Pharmacology, Faculty of Medicine, Al-Azhar University, Cairo 11511, Egypt; 8 Department of Anatomy, College of Medicine, King Khalid University, Abha 61421, Saudi Arabia; tarigfuture@gmail.com; 9 Department of Basic Medical Science, College of Medicine, Imam Mohammad Ibn Saud Islamic University, Riyadh 11432, Saudi Arabia; haabdrabou@imamu.edu.sa; 10 Department of Basic Medical Science, Dar Al-Uloom University, Riyadh 11512, Saudi Arabia; m.samhan@dau.edu.sa

**Keywords:** anti-inflammatory, atopic dermatitis, diclofenac, *Eclipta prostrata* (L.) L., haemolysis, non-steroidal anti-inflammatory drugs, synergic activity

## Abstract

Atopic dermatitis (AD) or eczema is an important inflammatory chronic skin disease that brings many complications in its management and treatment. Although several chemical agents are used for treatment, the search for better anti-inflammatory and antibacterial agents of plant origin has been ongoing, since natural compounds, it is commonly believed, are less dangerous than synthetic ones. Therefore, the present study explored a medicinal plant—*Eclipta prostrata* (L.) L.—for its anti-inflammatory activity alone and in combination with a non-steroidal anti-inflammatory drug (NSAID), diclofenac. The plant extract was used to make a cream formulation for treating atopic dermatitis and as an antibacterial agent against *Staphylococcus aures*, the major infectious agent associated with AD. The phytochemical analysis of the *E. prostrata* extract showed the presence of various phytochemicals, including flavonoids, Tannin, saponin, terpenoids, glycosides, phenol, alkaloids, quinone, and protein. The GC-MS profiling of methanolic *E. prostrata* extract was performed predicted the presence of twenty important phytochemicals, including 2-[5-(2-Hydroxypropyl) oxolan-2-yl]propanoic acid, dl-Menthol, dodecane, undecane, 4,7-dimethyl-, dodecane, 2,6,10-trimethyl-, decane, 2,3,5,8-tetramethyl-, cholest-5-en-3-ol, (3.alpha.)-, TMS derivative, cyclopropane carboxylic acid, 1-hydroxy-, (2,6-di-t-butyl-4-methylphenyl) ester, alpha.-farnesene, propanoic acid, 2-methyl-, 2-ethyl-1-propyl-1,3-propanediyl ester, diethyl phthalate, corticosterone, 2-methylpropionate, hentriacontan-13-ol, O-TMS, phthalic acid, 2,4-dimethylpent-3-yl dodecyl ester, hexasiloxane, 1,1,3,3,5,5,7,7,9,9,11,11-dodecamethyl-, acetic acid, 4-t-butyl-4-hydroxy-1,5-dimethyl-hex-2-ynyl ester, octadecane, 2-methyl- octacosane, 1-iodo-, nonacosane, and eicosyl isopropyl ether. Using an egg albumin denaturation inhibition assay, the anti-inflammatory activities of *E. prostrata* alone and in combination with diclofenac were investigated, and they showed 93% and 99% denaturation inhibition at 5 mg concentration of *E. prostrata* in alone and combination with diclofenac, respectively. Heat-induced haemolysis showed 2.5% and 2.4% of haemolysis at 5 mg of *E. prostrata* alone and in combination with diclofenac, respectively. An MTT assay performed using L_929_ cells proved that the extract has no cytotoxic effect. The plant extract displayed potential antibacterial activity against *Staphylococcus aureus*; the growth was inhibited at 1 mg/mL of *E. prostrata* extract. Thus, based on this evidence, the authors suggest that *E. prostrata* extract should be studied further for its anti-inflammatory and antibacterial activities and topical application in the treatment of atopic dermatitis.

## 1. Introduction

Skin, the largest organ in the human body and which covers the entire body surface and accounts for around up to 15% of total body weight, provides specific functions like protection, restraint, transpiration, thermal regulation, homeostasis, and sensitivity [1,2,3,4], etc., and has three layers including the epidermis, the dermis, and the hypodermis [5,6]. Although the layers act like a barrier, skin infections are predominant in all age groups, which may be due to exposure to radiation, bacteria, biological toxins, and natural chemical agents [7,8,9]. In addition, during the summer, the skin becomes dehydrated due to extreme heat, which causes pigmentation, sunburns, spots, marks, and wrinkles, and in extreme winter, the skin may form cuts, cracks, lacerations, and infections [10,11,12]. As a result, skin tissue alteration can happen, leading to skin ageing and skin problems, resulting in the occurrence of an inflammatory response [13,14]. Inflammation is a significant and essential component of the innate immune system, and it is a primary response to any harmful stimuli, including infection and injury, leading to pathogen elimination and the removal of infected sites [15,16]. Although potent non-steroidal anti-inflammatory drugs (NSAIDs) are used for the treatment of inflammatory conditions, their adverse effects on gastrointestinal, cardiovascular, hepatic, renal, cerebral, and pulmonary systems, resulting in organ damage [17,18], triggered a search for new alternatives for these ‘wonder drugs’.

Medicinal plants and plant-based formulations have been used for centuries in traditional medicine, and due to their capability to target numerous signalling pathways with minimal side effects, minimum damage to the tissues, and accommodation of better comfort to the patients [19,20,21,22], they have currently been receiving more attention. *Eclipta prostrata* (L.) L. from *Asteraceae* family is a medium-sized branched medicinal herb that bears white flowers found in tropical and subtropical regions of the world [23,24]. Traditionally, it has been used for treating various skin problems such as wounds, hair loss prevention, and dermatitis, owing to the presence of secondary metabolites, including thiophene derivatives, triterpenes, polyacetylenes, steroids, coumestans, and polypeptides [25,26]. Therefore, the present study investigates the anti-inflammatory activities of the methanolic *E. prostrata* extracts alone and in combination with an NSAID, diclofenac. In addition, *E. prostrata* has also been analysed for its antibacterial activity against *Staphylococcus aureus,* which is predominantly involved in atopic dermatitis.

## 2. Materials and Methods

### 2.1. Collection of Plants and Their Extraction

The collected *Eclipta prostrata* plant from a nursery yard in Al-Kharj, Saudi Arabia, was cleaned with tap water to remove the dust, and the leaves were air-dried and finely powdered. In total, 20 g of the powder was added into the cellulose thimble and kept in the Soxhlet apparatus. The reaction started once the methanol was added and the reaction continued for several hours at 60 °C until a clear methanol solution was obtained. The attained final product after solvent evaporation was used for further analysis [27].

### 2.2. Phytochemical Screening of the Eclipta prostrata Extract

To investigate the presence of preliminary phytochemicals such as proteins, amino acids, volatile and essential oils, tannins, steroids, carbohydrates, glycosides, and alkaloids, various screening tests were conducted, including protein detection using a xanthoproteic assay, saponin identification (foam test, steroid test, terpenoids, alkaloids, and phenol and tannin test), a test for flavonoids (Shinoda test, alkaline regent test, and glycoside test), etc., on *E. prostrata* using standard protocols [28,29,30,31,32].

### 2.3. Identification of Bioactive Metabolites in the Extract Using GCMS

The bioactive compounds in the prepared crude extract of *E. prostrata* were analysed using gas chromatography and mass spectrum (GC-MS, Shimadzu, Nexis GC-2030, Kyoto, Japan) [33]. Before sample injection, all the required programmes were initiated, such as temperature, gas, etc., and the scanning was continued for 30 min. The compounds eluted from the column were detected and the separated compounds were signified as peaks, and each peak was denoted as a single compound in the chromatogram which was generated using the mass spectroscopy detector. Then, the separated compounds were identified based on the mass spectra patterns and retention indices that existed in the library.

### 2.4. Egg Albumin Denaturation Inhibition Assay for the Extract

To evaluate the anti-inflammatory properties of *E. prostrata* alone and in combination with the drug diclofenac, an egg albumin denaturation assay was performed as per the standard protocol [34]. Briefly, the various concentrations of *E. prostrata* (5 mg/mL, 10 mg/mL, 20 mg/mL, 25 mg/mL, and 30 mg/mL) alone and *E. prostrata* (5 mg/mL, 10 mg/mL, 20 mg/mL, 25 mg/mL, and 30 mg/mL) in combination with diclofenac (5 mg/mL, 10 mg/mL, 20 mg/mL, 25 mg/mL, and 30 mg/mL) (1:1) were added to the egg albumin and phosphate-buffered saline (PBS) at pH 6.4, and all the mixtures were incubated for 10 min at 37 °C followed by heating at 70 °C for another 20 min in a water bath to stimulate the egg albumin denaturation. The denaturation of each sample was measured at 660 nm using a spectrophotometer. The protein denaturation inhibition percentage was calculated using the following formula:

% inhibition = (Control OD − Sample OD)/Control OD × 100

### 2.5. Heat-Induced Haemolysis

The extract of *E. prostrata* alone and combined with the diclofenac were examined for their effects on red blood cell membranes using a haemolysis process induced by heat [35]. In brief, several concentrations of *E. prostrata* (5 mg/mL, 10 mg/mL, 20 mg/mL, 25 mg/mL, and 30 mg/mL) in combination with diclofenac (5 mg/mL, 10 mg/mL, 20 mg/mL, 25 mg/mL, and 30 mg/mL) (1:1) were added to previously prepared 2% RBC (1 mL) suspension. The mixture was kept in a water bath at 56 °C for 30 min, and the mixture was centrifuged at 2000 rpm for 10 min, and the obtained supernatant was measured at 560 nm. The percentage of haemolysis was calculated using the following formula:

% of haemolysis = [(Sample OD − negative control OD)/(Positive control OD − negative control OD)] × 100

### 2.6. Preparation of Anti-Inflammatory Cream Using the Extract

An anti-inflammatory cream was prepared using 15% of *E. prostrata* alone and *E. prostrata* in combination with diclofenac (1:1) using hot emulsification, as per standard protocol [36]. In the hot emulsification, the water and oil phases were melted separately at 70 °C, and the water phase was added to the oily phase (5% glycerol monostearate, 50% liquid paraffin, 5% stearic acid, 0.1% lauryl sulphate, 5% glycerol, absolute ethanol, and purified water 100 mL) after continuous stirring and vortexing of the mixture. Finally, it was mixed with methylparaben, and the obtained cream was used for further studies 

### 2.7. E. prostrata Methanolic Extract Cytotoxicity

Cytotoxicity analysis was carried out for *E. prostrata* methanolic extract on the L_929_ cell line (mouse fibroblast) using an MTT assay, as per the standard procedure [37]. Shortly, the L_929_ cells grown in Dulbecco’s Modified Eagles Medium (DMEM) with 10% foetalfetal bovine serum were treated with different concentrations (5, 10, 15, 20, and 30 mg/mL) for 24 h, followed by MTT solution addition to form formazan crystals. DMSO was added to dissolve crystals and measured at 570 nm to calculate the percentage of cell viability.

### 2.8. Antibacterial Activity of E. prostrata Methanolic Extract 

*E. prostrata* methanolic extract was studied for its antibacterial potential against *Staphylococcus aureus* using the well diffusion method [38]. Briefly, the *S. aureus* overnight culture adjusted for 0.5 MacFarland was swabbed on sterile BHI plates, and various concentrations of *E. prostrata* methanolic extract were added to the drilled well and incubated at 37 °C for 24 h. The inhibition of growth around the extract loaded well-indicated the antibacterial activity of *E. prostrata* methanolic extract against *S. aureus*. Here, methanol and tetracycline (3 µg/well) were used as vehicle and positive controls.

### 2.9. Determination of MIC for E. prostrata Methanolic Extract

The MIC of *E. prostrata* methanolic extract was investigated against *S. aureus* using the well diffusion method [37]. In brief, the serially diluted 4 mg/mL of *E. prostrata* methanolic extract was finally made to 0.03 mg/mL in BHI broth, followed by the addition of overnight *S. aureus* culture and incubation. Then, the turbidity of each well was measured at 600 nm.

### 2.10. Statistical Analysis

The error bar was calculated from the mean and standard deviations of the egg albumin denaturation assay, heat-induced haemolysis, cytotoxicity, and MIC determinations.

## 3. Results

### 3.1. Phytochemical Screening of E. prostrata Methanolic Extract

The secondary metabolites present in the methanolic extract of *E. prostrata* are presented in Table 1. As shown in the table, *E. prostrata* has been reported to have tannin, flavonoids, saponin, terpenoids, glycosides, phenol, alkaloids, quinone, and protein. Here, the extract was not reported for steroids.

### 3.2. GC-MS Profiling of E. prostrata Methanolic Extract

**Figure 1:**
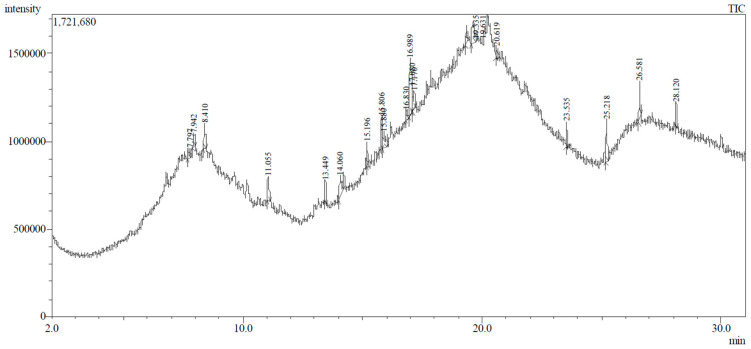
GC-MS chemical profiling of *E. prostrata* in methanolic fraction. TIC = tentatively identified compounds); min = minutes.

The chemical profiling of *E. prostrata* in the methanolic fraction was analysed to find the different chemical compounds, and the identified individual compounds from the methanolic extract are presented in Figure 1. The mass fragmentation pattern was used to analyse the compound structures, and the chemical profile was compared with spectral data existing in the National Institute of Standards and Technology (NIST) library. As noted in the figure, the chromatogram present in the methanolic extract showed twenty important phytochemicals, including 2-[5-(2-hydroxypropyl) oxolan-2-yl]propanoic acid, dl-menthol, dodecane, undecane, 4,7-dimethyl-, dodecane, 2,6,10-trimethyl-, decane, 2,3,5,8-tetramethyl-, cholest-5-en-3-ol, (3.alpha.)-, TMS derivative, cyclopropanecarboxylic acid, 1-hydroxy-, (2,6-di-t-butyl-4-methylphenyl) ester, alpha.-farnesene, propanoic acid, 2-methyl-, 2-ethyl-1-propyl-1,3-propanediyl ester, diethyl phthalate, corticosterone, 2-methylpropionate, hentriacontan-13-ol, O-TMS, phthalic acid, 2,4-dimethylpent-3-yl dodecyl ester, hexasiloxane, 1,1,3,3,5,5,7,7,9,9,11,11-dodecamethyl-, acetic acid, 4-t-butyl-4-hydroxy-1,5-dimethyl-hex-2-ynyl ester, octadecane, 2-methyl- octacosane, 1-iodo-, nonacosane, and eicosyl isopropyl ether. In addition, retention time, peak area, percentage peak area, height percentage, and names of identified compounds are displayed in Table 2.

### 3.3. Egg Albumin Denaturation Inhibition Assay of the Extract

**Figure 2:**
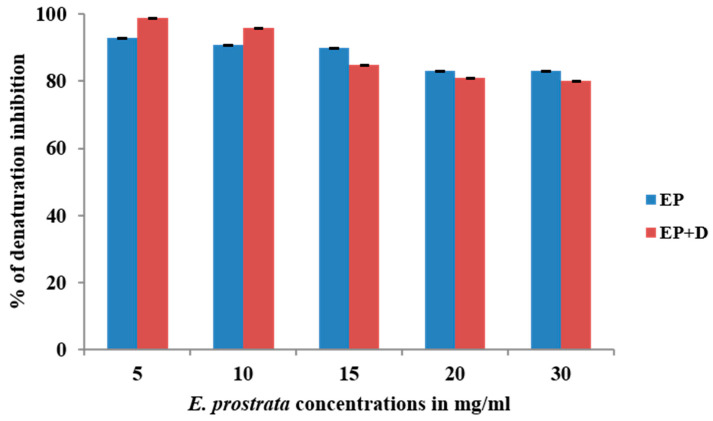
Inhibition percentage of *E. prostrata* methanolic extract alone and in combination with diclofenac on egg albumin denaturation. EP = *E. prostrata,* EP + D = *E. prostrata* + diclofenac.

The anti-inflammatory properties of *E. prostrata* alone and in combination with diclofenac on egg albumin denaturation were evaluated, and the obtained percentage of egg albumin denaturation inhibition is presented in Figure 2. The graph represents the inhibiting ability of different concentrations of *E. prostrata* (5 mg/mL, 10 mg/mL, 20 mg/mL, 25 mg/mL, and 30 mg/mL) alone and *E. prostrata* in combination with diclofenac (1:1) on egg albumin denaturation. As noted in the graph, *E. prostrata* methanolic extract different concentrations inhibited 93%, 91%, 90%, 83%, and 83% egg albumin denaturation. Similarly, *E. prostrata* combination with diclofenac (1:1) inhibited 99%, 96%, 85%, 81%, and 80% egg albumin denaturation, indicating anti-inflammatory properties.

### 3.4. Heat-Induced Haemolysis

**Figure 3:**
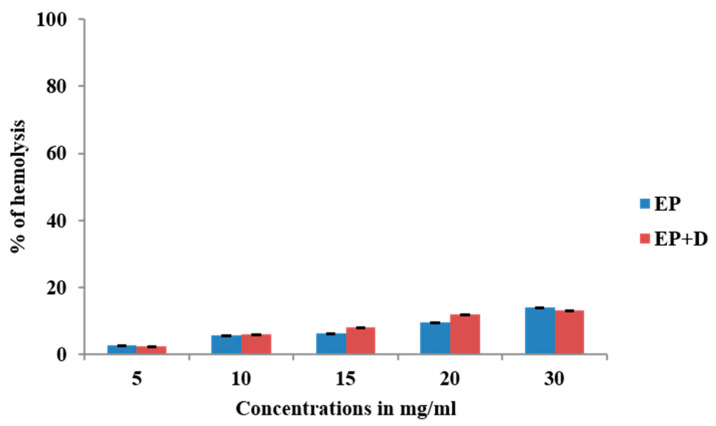
Heat-inducing hemolysishaemolysis percentage of *E. prostrata* methanolic extract alone and in combination with diclofenac. EP = *E. prostrata,* EP + D = *E. prostrata* + diclofenac.

The ability of *E. prostrata* methanolic extract alone and in combination with diclofenac on red blood cell membrane was investigated by inducing heat, and the attained percentage of haemolysis is displayed in Figure 3. As represented in the figure, varying concentrations of *E. prostrata* methanolic extract (5 mg/mL, 10 mg/mL, 20 mg/mL, 25 mg/mL, and 30 mg/mL) showed 2.5%, 5.5%, 6.2%, 9.5%, and 14% haemolysis, respectively. Similarly, *E. prostrata* combination with diclofenac (1:1) showed 2.4%, 6%, 8%,12%, and 13% haemolysis.

### 3.5. E. prostrata Methanolic Extract Cream and Its Cytotoxicity Analysis

**Figure 4:**
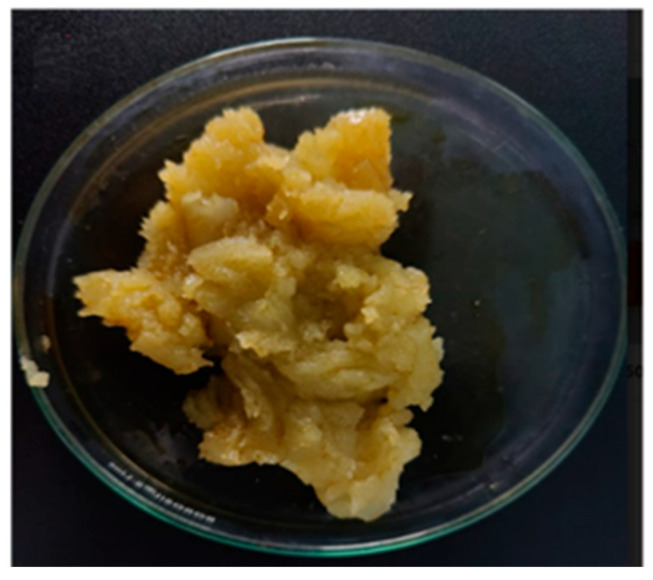
*E. prostrata* 15% methanolic extract cream effect on human skin prepared.

**Figure 5:**
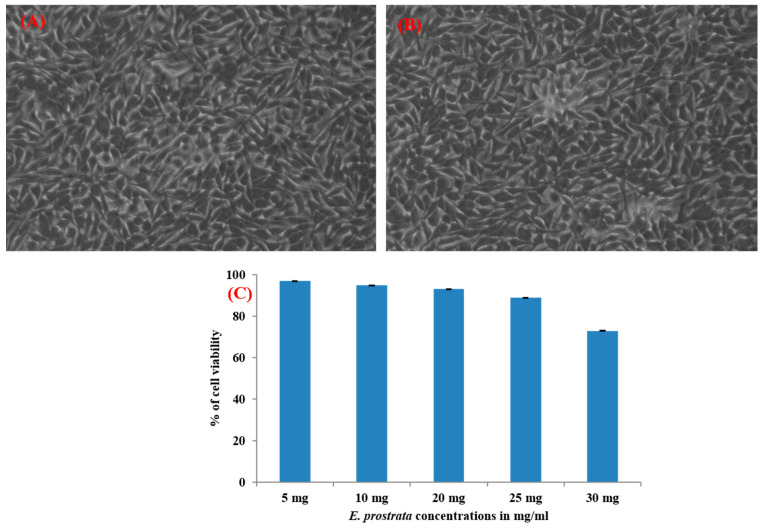
Cell viability percentage of *E. prostrata* methanolic extract after treating L_929_ cells using MTT assay. (**A**) Untreated L_929_ cell morphology. (**B**) L_929_ cell morphology after treatment with *E. prostrata*. (**C**) The graph represents the percentage of cell viability after treatment.

The prepared *E. prostrata* methanolic extract cream was prepared (Figure 4), and its effect on L_929_ cells was studied using an MTT assay. The calculated cell viability percentage after treatment with varying concentrations of *E. prostrata* methanolic extract is presented in Figure 5.

As seen in the plotted graph, the L_929_ cells treated with a wide range of extract concentrations as 5 mg/mL, 10 mg/mL, 20 mg/mL, 25 mg/mL, and 30 mg/mL showed 97%, 95%, 93%, 89%, and 73% cell viability when compared to untreated cells.

### 3.6. Antibacterial Activity of E. prostrata Methanolic Extract

**Figure 6:**
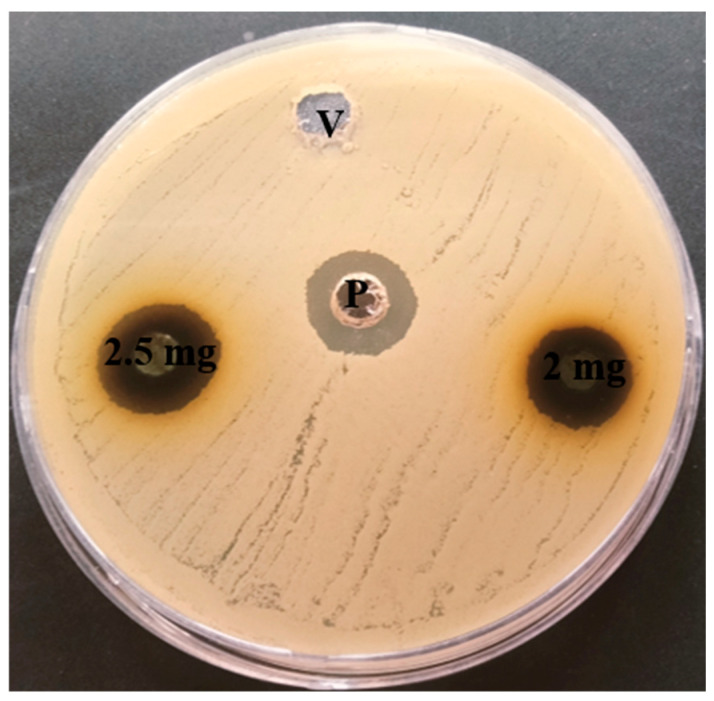
*E. prostrata* methanolic extract antibacterial activity against *S. aureus.* Two different concentrations exhibited inhibition zones around the well. Note: P—positive control (tetracycline) and v—vehicle control.

The *E. prostrata* methanolic extract antibacterial activity was explored against *S. aureus* through the well diffusion method, and the observed growth inhibition is represented in Figure 6. As seen in the figure, two different concentrations (2 mg/well and 2.5 mg/well) of *E. prostrata* methanolic extract showed zone inhibitions of 14 mm and 15 mm, respectively. The results reveal that *E. prostrata* methanolic extract’s antibacterial activity was dose-dependent.

### 3.7. E. prostrata Methanolic Extract MIC Determination

**Figure 7:**
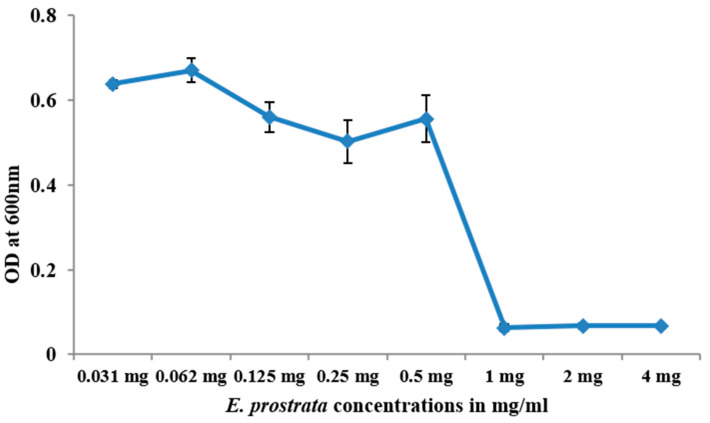
*E. prostrata* methanolic extract MIC was determined against *S. aureus*, and the growth inhibition was noted at 1 mg/mL.

The *E. prostrata* methanolic extract MIC was determined against *S. aureus,* and the calculated *E. prostrata* with the least growth inhibitory concentration plotted against *S. aureus* is displayed in Figure 7. As shown in the figure, the graph was plotted against concentrations vs. optical density, representing the *E. prostrata* 1 mg/mL of concentrations needed to inhibit the growth of *S. aureus.*

## 4. Discussion

Atopic dermatitis is one of the most important chronic skin diseases that affect the skin, resulting in high itchy inflammation, common eczematous lesions, and fluctuation. However, the existing treatment contains a multi-stage method that aims to create persistent disease control to improve the quality of a patient’s life. As most of the NSAIDs used in the treatment of atopic dermatitis are known to cause serious side effects, the researchers are concentrating on inflammatory skin diseases and against microbes responsible for skin disease. Hence, the medicinal plant was used for skin diseases caused by microbial infection and inflammation [39,40]. Therefore, the present study focused on one of the medicinal plants, *E. prostrata,* and its methanolic extract was screened for its anti-inflammatory potential and antibacterial activities against *S. aureus*, which is involved in atopic dermatitis. Here, *E. prostrata* methanolic extract was prepared and their phytochemicals, such as flavonoids, tannin, saponin, terphenoid, glycosides, phenol, alkaloids, quinone, and protein, were identified, and the chemical profiling of *E. prostrata* in methanolic fraction revealed the presence of twenty individual compounds mainly responsible for *E. prostrata* anti-inflammatory and antibacterial activities. Many studies have proven that *E. prostrata* L. extract anti-inflammatory activities and their major biological components [41]. In support of this, a methanolic extract of *E. prostrata* revealed the anti-inflammatory activity through inhibition of NF-κB activation in the presence of chief components such as coumestans demethylwedelolactone and wedelolactone, which showed anti-inflammatory activity in acute asthma models (Morel et al., 2024) [42]. When the effects and molecular mechanisms of ethanolic extracts of *E. prostrata* were elucidated in a house dust mite (HDM)-induced AD in mice model, it was found that *E. prostrata* decreased the epidermis/dermis thickness, restored skin barrier dysfunction, infiltrated immune cells and imbalanced immune response, and also suppressed the phosphorylation of extracellular signal-regulated kinase/signal transducer and T helper (Th)1, Th2, and Th17 cytokines’ expressions in HDM-induced AD mice (Kang et al., 2022) [43]. A study that aimed to understand the anti-inflammatory potential of the *E. prostrata* methanolic leaf extract in carrageenan and egg white-induced hind paw oedema in rats also showed a dose-dependent activity [44].

Similarly, a recent study explored the different species of E. alba’s anti-inflammatory, antimicrobial, antimelanogenic, and antioxidant properties, as well as the presence of phytochemicals associated with treating various skin diseases and conditions. Important phytochemicals such as wedelolactone, luteolin, demethylwedelolactone, and luteolin-7-*O*-glucoside are responsible for anti-inflammatory activity, as well as eclalbasaponin and wedelolactone, which are reported to have antimicrobial activity on *E. alba* [45,46].

In addition, the anti-inflammatory activity of *E. prostrata* methanolic extract was investigated through egg albumin denaturation inhibition and haemolysis. In egg albumin denaturation, the protein structures (secondary and tertiary structures) have been disturbed due to various external components like stress, acid or base, organic or inorganic substances, and heat; also, the denaturation process leads to functional activity loss resulting in inflammation [47]. An egg albumin denaturation assay was used to measure the compounds’ ability to prevent the denaturation of egg albumin; thereby, its anti-inflammatory activity was evaluated. The compounds that have anti-inflammatory activity can able to stabilise the structural protein and prevent protein denaturation, which is always related to inflammation and results in tissue damage. Apparently, the compound that decreases the denaturation might have possible anti-inflammatory properties [48,49]. Our study exhibited the anti-inflammatory activity by inhibiting egg albumin denaturation; thereby, its anti-inflammatory activity was proved. In addition, in vitro haemolysis is the most employed toxicity test used to analyse the initial toxicity assessment during drug development, and it is related to cytotoxicity, which is mainly associated with cell membrane disruption [50]. The present study demonstrated no haemolysis after exposing *E. prostrata* methanolic extract to red blood cells, which indicated no toxicity towards cell membranes. In addition, a recent study reported the in vivo anti-inflammatory activity of diclofenac sodium, prednisolone, and atorvastatin when used in combination with ascorbic acid using Wistar Rats and found the highest inhibitory effect in a diclofenac and ascorbic acid combination, which suggests that diclofenac and the ascorbic acid combination was more effective against neuropsychiatric effects and local oedema caused by inflammation [51].

In addition, our study employed the prepared *E. prostrata* cream for a skin irritation test. Skin sensitivity is commonly increasing and is characterised by particular subjective discomfort complaints such as sensations of pain, tightness, stinging, burning, and erythema. Skin sensitivity may be activated by hypersensitivity to a variety of stimuli that could be physical, chemical, hormonal, or psychological [52,53]. Generally, the face is the most common skin-sensitive area, followed by the hands and scalp [54]. The present study investigated the prepared *E. prostrata* cream for skin irritation and found that no discomfort was observed on human skin, indicating that prepared *E. prostrata* cream may be effective or useful for skin application.

The antibacterial activities of *E. prostrata* methanolic extract were investigated against *S. aureus,* which is involved in atopic dermatitis. Our study investigated the antibacterial activity against *S. aureus* with minimal inhibitory concentration. In support of this, *E. prostrata* were evaluated for their antibacterial activity against *Staphylococcus aureus*, *Pseudomonas aeruginosa*, *Escherichia coli*, and *Klebsiella pneumoniae*, along with some other medicinal plants. The various studies demonstrated the antibacterial activity against all the tested pathogens with a high range of activity [55]. The various solvents, such as butanol and water-extracted *E. alba* extract, showed activity against *Bacillus* cereus, and ethyl acetate fraction exhibited activity against *B. subtilis* at 3 mg/disc. In the same way, the methanolic extract displayed activity against *Candida albicans* at 3 mg/disc and showed 60% zone of inhibition [56]. Similarly, the ethanolic extract of *E. alba* plant showed antibacterial properties against *S. aureus, Pseudomonas aeruginosa, Shigella flexneri*, and *Escherichia coli* at 1 mg/mL, and was also active against *Aspergillus niger* at 4 mg/mL, which showed 51.52% of inhibition [57]. Altogether, collectively, *E. prostrata* methanolic extract and its cream formulation have anti-inflammatory activity and may be used as potent therapeutic agents against atopic dermatitis.

## 5. Conclusions

As most of the anti-inflammatory chemical agents, including NSAIDs, used in the treatment of skin inflammations have many adverse side effects, the scientific community is in search of new anti-inflammatory agents with novel modes of action, and medicinal plants are one of the potential options. Therefore, the present study investigated the anti-inflammatory and antibacterial activities of one of such plants, *E. prostrata* (L.) L., and its methanolic extracts showed promising activities when analysed using an egg albumin denaturation assay and various antibacterial activity testing methods against *S. aureus*. The haemolysis and cytotoxicity analyses showed that the *E. prostrata* methanolic extract cream has no harmful effects on the cell membrane, thus primarily qualifying it to be used on human cells. Therefore, this investigation recommends further studies on the use of *E. prostrata* methanolic extract cream as an alternate anti-inflammatory and antibacterial agent in diseases like atopic dermatitis.

## Figures and Tables

**Table 1:** Phytochemical analysis of the *E. prostrata* methanolic extract.

Sl. No.	Phytochemicals	Results
1.	Tannin	Positive
2.	Flavonoids	Positive
3.	Saponin	Positive
4.	Steroid	Negative
5.	Terpenoid	Positive
6.	Glycosides	Positive
7.	Phenol	Positive
8.	Alkaloids	Positive
9.	Quinone	Positive
10.	Protein	Positive

**Table 2:** The presence of phytochemicals in methanolic fractions of *E. prostrata* using GC-MS.

Peak No.	Retention Time	Peak Area%	Height%	Name of the Compound	Structure
1	7.797	2.65	2.14	2-[5-(2-Hydroxypropyl)oxolan-2-yl]propanoic acid	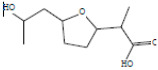
2	7.942	2.52	2.87	dl-Menthol	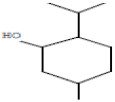
3	8.410	4.78	5.19	Dodecane	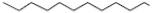
4	11.055	5.69	5.06	Undecane, 4,7-dimethyl-	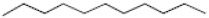
5	13.449	2.39	4.58	Dodecane, 2,6,10-trimethyl-	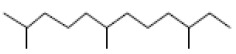
6	14.060	7.86	4.64	Decane, 2,3,5,8-tetramethyl-	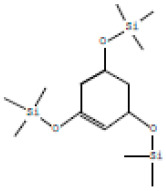
7	15.196	2.58	4.83	Cholest-5-en-3-ol, (3alpha.)-, TMS derivative	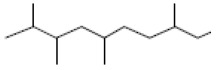
8	15.806	5.71	6.95	Cyclopropanecarboxylic acid, 1-hydroxy-, (2,6-di-t-butyl-4-methylphenyl) ester	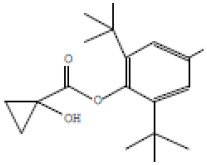
9	15.880	5.63	3.48	alpha-Farnesene	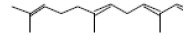
10	16.830	2.34	1.95	Propanoic acid, 2-methyl-, 2-ethyl-1-propyl-1,3-propanediyl ester	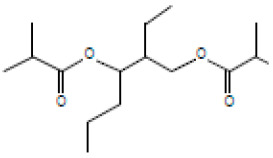
11	16.989	16.20	11.92	Diethyl Phthalate	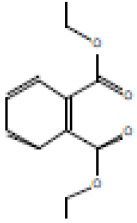
12	17.080	2.20	5.25	Corticosterone, 2-methylpropionate	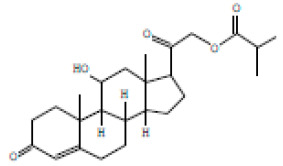
13	17.170	3.57	3.57	Hentriacontan-13-ol, O-TMS	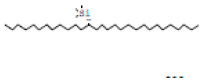
14	19.335	2.54	3.66	Phthalic acid, 2,4-dimethylpent-3-yl dodecyl ester	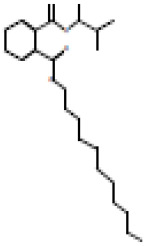
15	19.631	8.34	4.32	Hexasiloxane, 1,1,3,3,5,5,7,7,9,9,11,11-dodecamethyl-	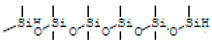
16	20.619	2.62	2.57	Acetic acid, 4-t-butyl-4-hydroxy-1,5-dimethyl-hex-2-ynyl ester	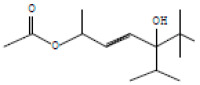
17	23.535	5.02	5.36	Octadecane, 2-methyl-	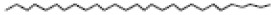
18	25.218	8.63	8.69	Octacosane, 1-iodo-	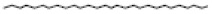
19	26.581	5.78	7.84	Onacosane	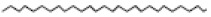
20	28.120	2.95	5.14	Eicosyl isopropyl ether	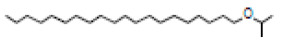

## Data Availability

The original contributions presented in this study are included in the article. Further inquiries can be directed to the corresponding authors.
